# *Populus euphratica* GRP2 Interacts with Target mRNAs to Negatively Regulate Salt Tolerance by Interfering with Photosynthesis, Na^+^, and ROS Homeostasis

**DOI:** 10.3390/ijms25042046

**Published:** 2024-02-07

**Authors:** Jing Li, Rui Zhao, Jian Liu, Jun Yao, Siyuan Ma, Kexin Yin, Ying Zhang, Zhe Liu, Caixia Yan, Nan Zhao, Xiaoyang Zhou, Shaoliang Chen

**Affiliations:** 1 State Key Laboratory of Efficient Production of Forest Resources, College of Biological Science and Technology, Beijing Forestry University, Beijing 100083, China; lijing70747@163.com (J.L.); ruizhao926@126.com (R.Z.); liujian20170703@163.com (J.L.); msyuan66@163.com (S.M.); ykx0303@126.com (K.Y.); zying@bjfu.edu.cn (Y.Z.); liuz6415@163.com (Z.L.); caixiayan2019@163.com (C.Y.); zhaonan19880921@126.com (N.Z.); zhouxiaoyang@bjfu.edu.cn (X.Z.); 2 Guangdong Provincial Key Laboratory of Silviculture, Protection and Utilization, Guangdong Academy of Forestry, Guangzhou 510520, China; yaojun990@126.com

**Keywords:** glycine-rich RNA-binding protein, RNA affinity purification sequencing, Na^+^ flux, ROS, antioxidant enzyme, photosynthesis, ATPase

## Abstract

The transcription of glycine-rich RNA-binding protein 2 (*PeGRP2*) transiently increased in the roots and shoots of *Populus euphratica* (a salt-resistant poplar) upon initial salt exposure and tended to decrease after long-term NaCl stress (100 mM, 12 days). *PeGRP2* overexpression in the hybrid *Populus tremula* × *P. alba* ‘717-1B4’ (*P.* × *canescens*) increased its salt sensitivity, which was reflected in the plant’s growth and photosynthesis. PeGRP2 contains a conserved RNA recognition motif domain at the N-terminus, and RNA affinity purification (RAP) sequencing was developed to enrich the target mRNAs that physically interacted with PeGRP2 in *P.* × *canescens*. RAP sequencing combined with RT-qPCR revealed that NaCl decreased the transcripts of PeGRP2-interacting mRNAs encoding photosynthetic proteins, antioxidative enzymes, ATPases, and Na^+^/H^+^ antiporters in this transgenic poplar. Specifically, PeGRP2 negatively affected the stability of the target mRNAs encoding the photosynthetic proteins *PETC* and *RBCMT*; antioxidant enzymes *SOD[Mn]*, *CDSP32*, and *CYB1-2*; ATPases *AHA11*, *ACA8*, and *ACA9*; and the Na^+^/H^+^ antiporter *NHA1*. This resulted in (i) a greater reduction in Fv/Fm, YII, ETR, and Pn; (ii) less pronounced activation of antioxidative enzymes; and (iii) a reduced ability to maintain Na^+^ homeostasis in the transgenic poplars during long-term salt stress, leading to their lowered ability to tolerate salinity stress.

## 1. Introduction

Soil salinization poses an increasing threat to agricultural and forest productivity and environmental sustainability [1,2]. Fast-growing poplar species are economically important bioenergy resources and ecologically important for environmental conservation [3,4]. Enhancing the salinity tolerance of *Populus* for large-scale afforestation in salt-affected areas would enable sustainable bioenergy production [5]. The selection and identification of appropriate targets are essential for the genetic modification and molecular breeding of salt-resistant poplars [5]. The transcriptional regulation of salt-responsive genes in *Populus euphratica* (a salt-tolerant poplar species) is critical for these plants to mediate ionic and ROS homeostasis and for their adaptation to a salt environment [6,7,8,9,10,11,12,13,14,15,16,17]. Glycine-rich RNA-binding proteins (GRPs) interact with target mRNAs in the nucleus and regulate the processing and folding of the mRNAs [18,19,20]. In plants, GRPs have been found to be expressed in response to various environmental stresses [21,22,23,24,25,26,27,28,29,30,31,32,33,34,35,36,37,38,39,40,41]. In previous studies, several members of the GRP family in *Arabidopsis* and rice enhanced their cold and freezing tolerance [19,21,27,29,37,41,42,43]. *OsGRP4* in rice is involved in the plant’s response to high-temperature stress [44]. In *Camelina sativa*, the differential expression of *CsGRP2a*, *CsGRP2b*, and *CsGRP2c* under salt or drought stress has been identified [23]. The overexpression of *AtGRP2* or *AtGRP7* improves the drought tolerance and grain yield of rice [45]. The transcription of *SbGR-RNP* was increased twofold to fourfold in *Sorghum bicolor* seedlings treated with NaCl (0.5–1.0 M, 24 h) [46]. The *MhGR-RBP1* transcripts in the leaves of *Malus hupehensis* increased twofold after initial salt exposure (two days) but returned to the control levels after long-term treatment [24]. Although the salt induction of GRPs has been observed in herbaceous and woody species, GRPs can affect plants’ salt tolerance in negative or positive ways. AtGRP2 increased seed germination under salinity stress [19]. Similarly, the overexpression of *AtGRDP2* led to the upregulation of stress-responsive genes in lettuce plants [47]. However, the expression of *Zosia japonica ZjGRP* or *Medicago sativa MsGRP* inhibited seed germination and plant growth under salt stress [48,49]. Similarly, phenotype tests of the *AtGRP7* and *AtGR-RBP4* transgenic lines demonstrated that GRP7 and GR-RBP4 negatively affected germination under high salt levels [33,50]. However, the *Atgrp7* mutant rendered seedlings hypersensitive to NaCl [22]. It is unknown whether *P. euphratica PeGRP2* negatively or positively mediates plant growth, photosynthesis, and salt tolerance in poplar trees.

The salt tolerance of plants is related to their ability to regulate ionic and ROS homeostasis [1,2,5]. GRPs are involved in the mediation of the K^+^/Na^+^ balance and ROS production under salt stress. *Limonium bicolor LbGRP1* increased the salt tolerance of tobacco plants by increasing the proline content and the activity of catalase (CAT) and superoxide dismutase (SOD) [51]. Furthermore, *LbGRP1-* in transgenic tobacco was shown to limit the Na^+^ content and Na^+^/K^+^ ratio under saline conditions [51]. In addition, the overexpression of the *MpGR-RBP1* gene from *Malus prunifolia* decreased the salt-promoted ROS production in *Arabidopsis* [52]. In contrast, *ZjGRP* overexpression resulted in the downregulation of *SOD* and peroxidase (*POD*) genes in transgenic *Arabidopsis* [49]. In rice, OsGRP3 negatively regulated the expression of ROS regulatory genes, such as metallothionein 1d (*MT1d*) and peroxidase 1 (*POX1*), under water-deficient conditions [20]. AtGRP2 suppressed mitochondrial Mn-SOD and mitochondrial peroxiredoxin, whose function are related to controlling ROS homeostasis, under cold stress [19]. Whether PeGRP in *P. euphratica* interacts with target mRNAs to regulate ionic and ROS homeostasis is unclear.

This study aimed to evaluate whether *P. euphratica* PeGRP interacts with target mRNAs to regulate photosynthesis and ionic and ROS homeostasis under salt stress. We examined the NaCl-altered expression of *PeGRP2* in *P. euphratica* seedlings. Subsequently, the *PeGRP2* gene was cloned, and sequence analysis revealed that PeGRP2 resembled the GRPs of other species that contain a conserved RNA recognition motif domain at the N-terminus and interact with target mRNAs [18,20]. *PeGRP2* was then introduced into the hybrid poplar *Populus tremula* × *P. alba* ‘717-1B4’ (*P.* × *canescens*) to investigate the interaction between PeGRP2 and its target mRNAs. Phenotype tests showed that *PeGRP2* overexpression negatively affected the salt tolerance by reducing photosynthesis, increasing Na^+^ accumulation, and restricting ROS scavenging in the transgenic poplars. Here, we developed RNA affinity purification sequencing (RAP sequencing) to enrich the target mRNAs that physically interacted with PeGRP2 in *P.* × *canescens*. The PeGRP2-interacting mRNAs encoding proteins related to photosynthesis, antioxidant defense, and Na^+^ homeostasis were identified using RAP sequencing. RT-qPCR was used to examine the transcripts of the target mRNAs interacting with PeGRP2 in this transgenic poplar after NaCl treatment (0 or 100 mM, 15 days). The PeGRP2-targeting mRNA transcripts were also tested in the WT and served as salt and non-salt controls. Our RAP sequencing and RT-qPCR data showed that PeGRP2 negatively regulated the transcripts of several target mRNAs encoding proteins involved in photosynthesis, antioxidant defense, and Na^+^ homeostasis under saline conditions, thereby reducing transgenic *P.* × *canescens*’s ability to tolerate salinity. Our study provides new insights for the breeding of salt-resistant poplars by reducing the *GRP2* transcripts.

## 2. Results

### 2.1. Expression Profile of PeGRP2 in Salt-Stressed P. euphratica

**Figure 1:**
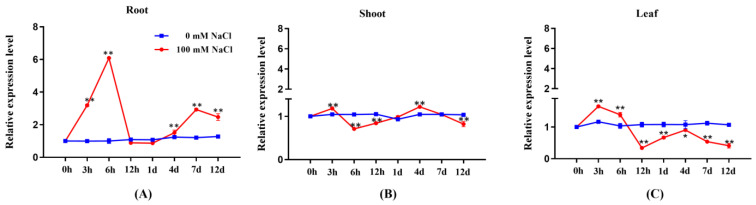
Transcription profile of *PeGRP2* in roots and shoots of *P. euphratica* under salt stress. Uniform seedlings of *P. euphratica* were treated with NaCl solution (0 or 100 mM) for 12 days. For RT-qPCR analysis, fine roots, shoots, and upper leaves (5th to 20th from shoot tip) were collected at day 1 (0 h, 3 h, 6 h, 12 h, 24 h), day 4, day 7, and day 12. (**A**) *PeGRP2* transcription in roots. (**B**) *PeGRP2* transcription in shoots. (**C**) *PeGRP2* transcription in leaves. The primer sequences for *PeGRP2* and the reference gene, *PeActin7*, are shown in Supplementary Table S1. Data are means ± SD (n = 3), and bars with asterisks indicate significant differences, *: *p <* 0.05, **: *p <* 0.01.

The transcription of *P. euphratica PeGRP2* fluctuated during the observation period under salt treatment (100 mM NaCl, 12 days). The *PeGRP2* expression in the roots tended to increase after the start of salt exposure and peaked at 6 h, followed by a rapid drop at 12 h (Figure 1A). Thereafter, the *PeGRP2* levels remained constant, other than the 1-fold increase that occurred at day 7 and day 12 (Figure 1A). The salt-altered *PeGRP2* transcription in the shoots and leaves resembled the trend in the roots, but the transient increase that occurred at 3 h was considerably lower than in the roots (Figure 1B,C). Furthermore, the *PeGRP2* expression in the shoots tended to decline after day 4 and returned to a level lower than that of control plants at day 12 (Figure 1B,C).

### 2.2. Sequence Analyses of PeGRP2

**Figure 2:**
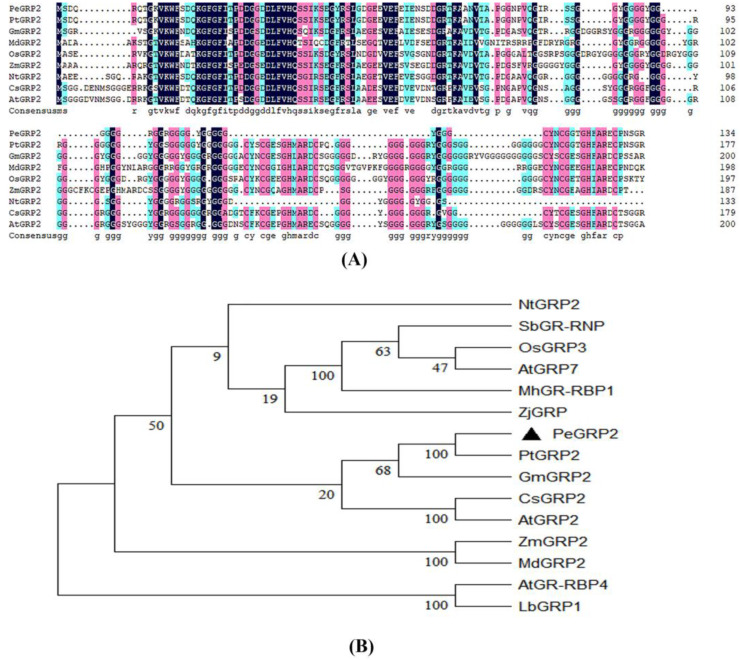
Multiple sequence alignment and phylogenetic analysis of PeGRP2. (**A**) Multiple sequence alignment between PeGRP2 and GRPs from other species. The GRP sequence of PeGRP2 was compared with those of different species, such as *Populus euphratica*, *Populus trichocarpa*, *Glycine max*, *Malus domestica*, *Oryza sativa*, *Zea mays*, *Nicotiana tabacum*, *Camelina sativa*, and *Arabidopsis thaliana*. Repeated amino acid sequences are shown in black, and other shadings represent conserved amino acids. (**B**) Phylogenetic analysis of GRPs from different species. The species are listed as follows: *Nicotiana tabacum*, *Sorghum bicolor*, *Oryza sativa*, *Arabidopsis thaliana*, *Malus hupehensis*, *Zoysia japonica*, *Populus euphratica*, *Populus trichocarpa*, *Glycine max*, *Camelina sativa*, *Zea mays*, *Malus domestica*, and *Limonium bicolor*. The target PeGRP2 is labelled with a black triangle (▲). The GRP accession numbers are shown in Supplementary Table S2.

The length of the *PeGRP2* sequence was 405 bp, coding for 134 amino acids, and the protein molecular weight was 13.44 kDa, with an isoelectric point of 5.15 (Figure 2A). The PeGRP2 protein was rich in glycine at the C-terminus, and the N-terminus contained a conserved RNA recognition motif (RRM) domain. The amino acid sequence of *P. euphratica* PeGRP2 resembled those of glycine-rich proteins from other species (Figure 2A). Phylogenetic tree analysis revealed that PeGRP2 was closely related to PtGRP2 from *P. trichocarpa* but distantly related to *Arabidopsis* AtGRP2 (Figure 2B).

### 2.3. Phenotypic Tests of PeGRP2-Overexpressing Poplars under Salt Stress

**Figure 3:**
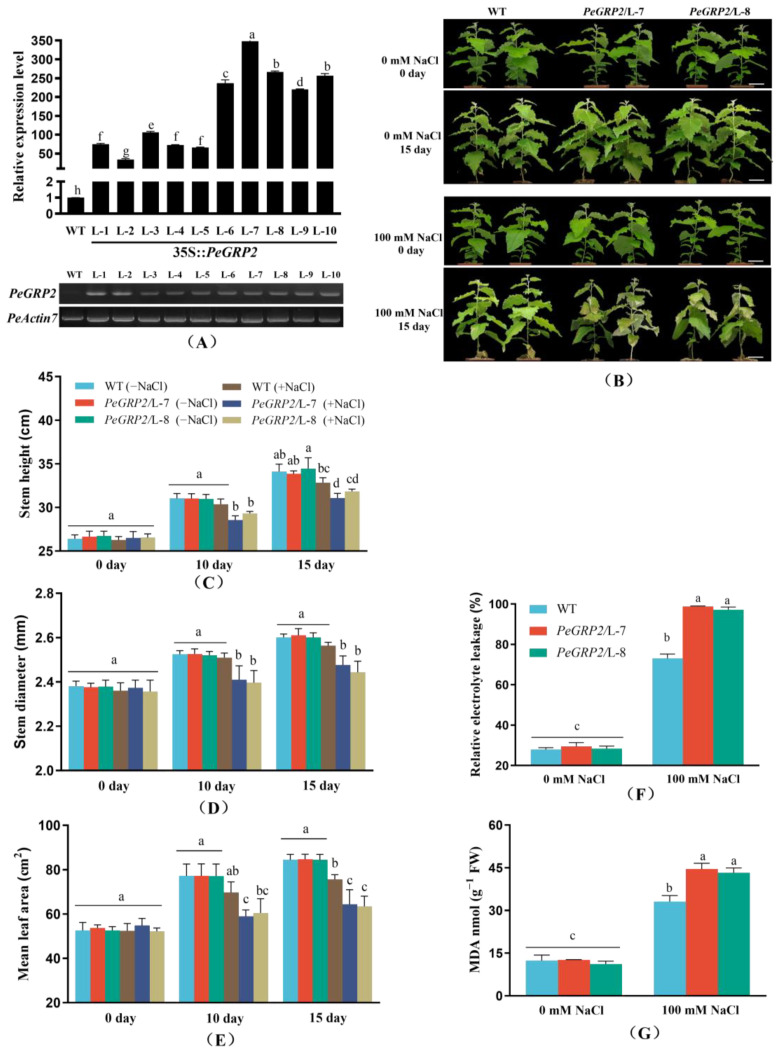
Phenotypic tests of wild-type *P.* × *canescens* and *PeGRP2*-overexpressing lines under long-term salt stress. (**A**) RT-qPCR and semi-quantitative PCR validation of *PeGRP2*. The primer sequences for *PeGRP2* and the reference gene, *PeActin7*, are shown in Supplementary Table S1. (**B**) Representative images showing plant performance of *PeGRP2*-overexpressing lines (L-7 and L-8) and wild-type (WT) *P.* × *canescens* after NaCl treatment with 0 or 100 mM for 15 days. Scale bars = 5 cm. (**C**) Stem height. (**D**) Stem diameter. (**E**) Mean leaf area. (**F**) Relative electrolyte leakage (REL). (**G**) Malondialdehyde content (MDA). After 15 days of NaCl (0 or 100 mM) treatment, mature leaves were sampled from WT and transgenic *P. × canescens* overexpressing *PeGRP2* (L-7 and L-8) to determine REL and MDA content. Data are means ± SD (n = 5), and bars with different letters indicate significant differences (*p* < 0.05).

*PeGRP2* was transformed into the gray poplar, *P. × canescens*, to determine the importance of *PeGRP2* in salinity tolerance. A total of 10 transgenic poplar lines (L-1, L-2, L-3, L-4, L-5, L-6, L-7, L-8, L-9, and L-10) were obtained, and the abundance of *PeGRP2* transcripts was verified using RT-qPCR and semiquantitative RT-PCR (Figure 3A). We selected a wild type (WT) and two transgenic lines with a higher *PeGRP2* abundance and plant growth, L-7 and L-8, for the salt tests (Figure 3A, Supplementary Figure S1). Under salt-free conditions, the shoot height, stem diameter, and leaf area of the *PeGRP2*-overexpressing poplars were comparable to those of the WT (Figure 3B–E). However, salt damage symptoms occurred in the mature upper leaves, with more severe damage in the transgenic lines (Figure 3B). The growth in height and diameter in the transgenic plants was more restricted under long-term salinity (100 mM NaCl, 10–15 days) than in the WT (Figure 3C,D). The reduced leaf area due to NaCl was also more pronounced in the transgenic plants compared to the WT (Figure 3E). This indicates that the overexpression of *PeGRP2* increases the salt sensitivity of *P.* × *canescens* poplars.

### 2.4. Membrane Permeability and Lipid Peroxidation

We measured the relative electrolyte leakage (REL) to observe whether the membrane permeability was increased by the salt treatment [7]. The NaCl treatment (100 mM, 15 days) caused a significant rise in the REL in the leaves, and the effect was more pronounced in the *P.* × *canescens* overexpressing *PeGRP2* (Figure 3F). The content of malondialdehyde (MDA) increased in the salt-treated plants, resembling the pattern of the REL (Figure 3G). This indicates that the increased REL due to salt stress in the transgenic lines results from lipid peroxidation, as MDA content is a free radical oxidative marker [7,53].

### 2.5. Photosynthetic Capacity of the Salinized Poplars

**Figure 4:**
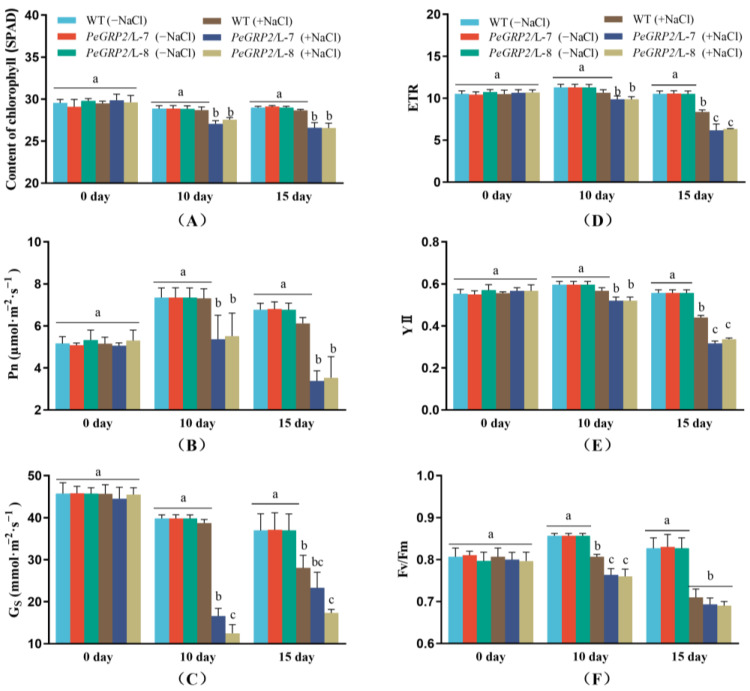
Effect of NaCl on chlorophyll content, photosynthesis, and fluorescence in wild-type *P.* × *canescens* and *PeGRP2*-overexpressing lines. (**A**) Chlorophyll content. (**B**) Net photosynthetic rate (Pn). (**C**) Stomatal conductance (Gs). (**D**) The relative electron transport rate (ETR). (**E**) The actual photosynthetic quantum yield (YII). (**F**) The maximum photochemical efficiency of PSII (Fv/Fm). The *PeGRP2*-overexpressing lines (L-7 and L-8) and wild-type (WT) *P. × canescens* were treated with NaCl saline (0 or 100 mM) for 15 days. Chlorophyll content, chlorophyll fluorescence (maximum photochemical efficiency of PSII, Fv/Fm; actual photosynthetic quantum yield, YII; and relative electron transport rate, ETR), and leaf gas exchange (net photosynthetic rate, Pn, and stomatal conductance, Gs) were examined on day 0, day 10, and day 15 in control and salinized plants. Data are means ± SD (n = 3), and bars with different letters indicate significant differences (*p* < 0.05).

Genotypic differences in growth performance are associated with the photosynthetic capacity of the WT and transgenic poplars. The chlorophyll content, chlorophyll fluorescence, and photosynthetic rate were examined in the NaCl-treated poplars. Salinity did not affect the chlorophyll content in the mature upper leaves of the WT poplars but significantly decreased the chlorophyll content in both transgenic lines L-7 and L-8 during the salt treatment (10–15 days, Figure 4A). Compared to the WT, the Pn in L-7 and L-8 started to decrease after 10 days of salt exposure, and a sharp decrease occurred on day 15 (Figure 4B), coinciding with a marked decrease in the stomatal conductance (Gs) in the leaves (Figure 4C). The chlorophyll fluorescence assay showed that NaCl reduced the maximum PSⅡ photochemical efficiency (Fv/Fm), the actual photosynthetic quantum yield (YⅡ), and the relative electron transport rate (ETR) in the WT poplars and the transgenic lines (Figure 4D–F). Compared with the WT, L-7 and L-8 showed a greater decrease in ETR, YII, and Fv/Fm after 10 and 15 days of salt stress (Figure 4D–F).

### 2.6. Activity of Antioxidant Enzymes

**Figure 5:**
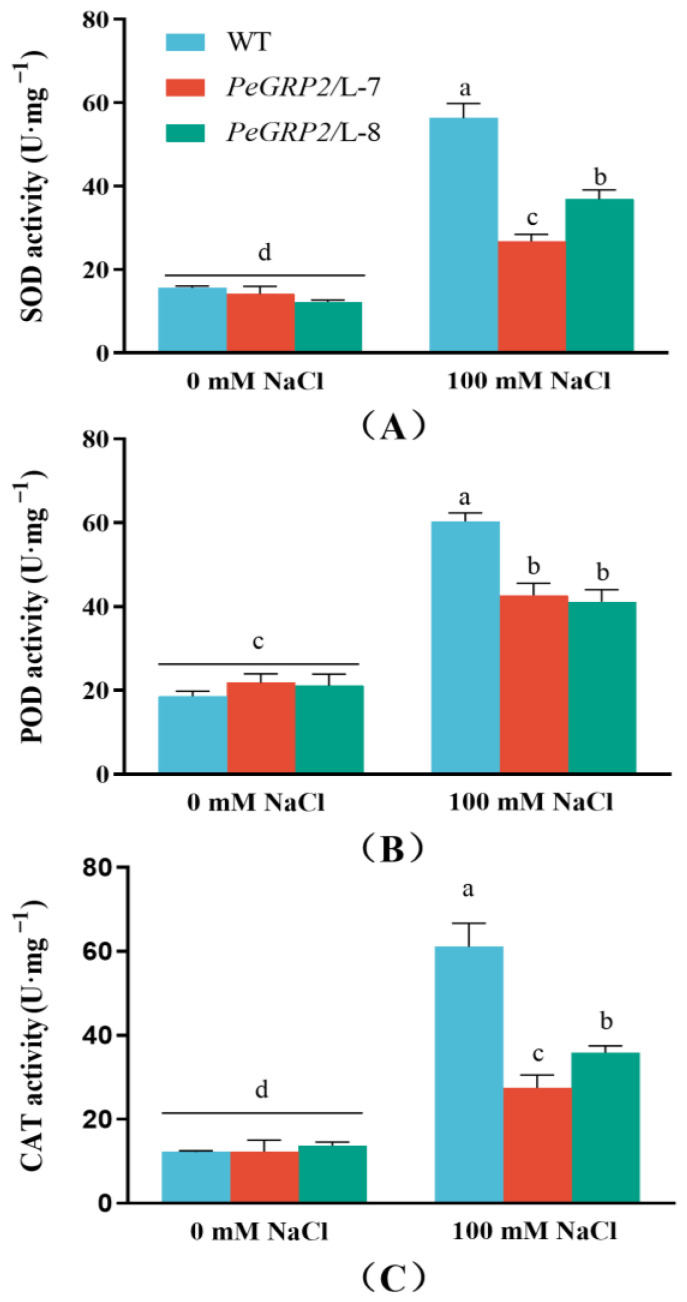
Antioxidant enzyme activity in wild-type *P.* × *canescens* and *PeGRP2*-overexpressing lines under long-term salt stress. (**A**) SOD activity. (**B**) POD activity. (**C**) CAT activity. The *PeGRP2*-overexpressing lines (L-7 and L-8) and wild-type (WT) *P. × canescens* were treated with NaCl saline (0 or 100 mM) for 15 days. The activity of superoxide dismutase (SOD), peroxidase (POD), and catalase (CAT) was measured in the leaves of no-salt control and salinized plants. Data are means ± SD (n = 3), and bars with different letters indicate significant differences (*p* < 0.05).

To determine whether the *PeGRP2*-transgenic poplars differed from the WT in terms of ROS scavenging, the POD, SOD, and CAT activity was compared under salt stress. NaCl treatment (100 mM, 15 days) increased the total activity of the tested antioxidative enzymes in the WT *P.* × *canescens* and *PeGRP2*-overexpressing lines, but significantly lower values were observed in L-7 and L-8 (Figure 5A-C).

### 2.7. Na^+^ Content and Flux in Roots

**Figure 6:**
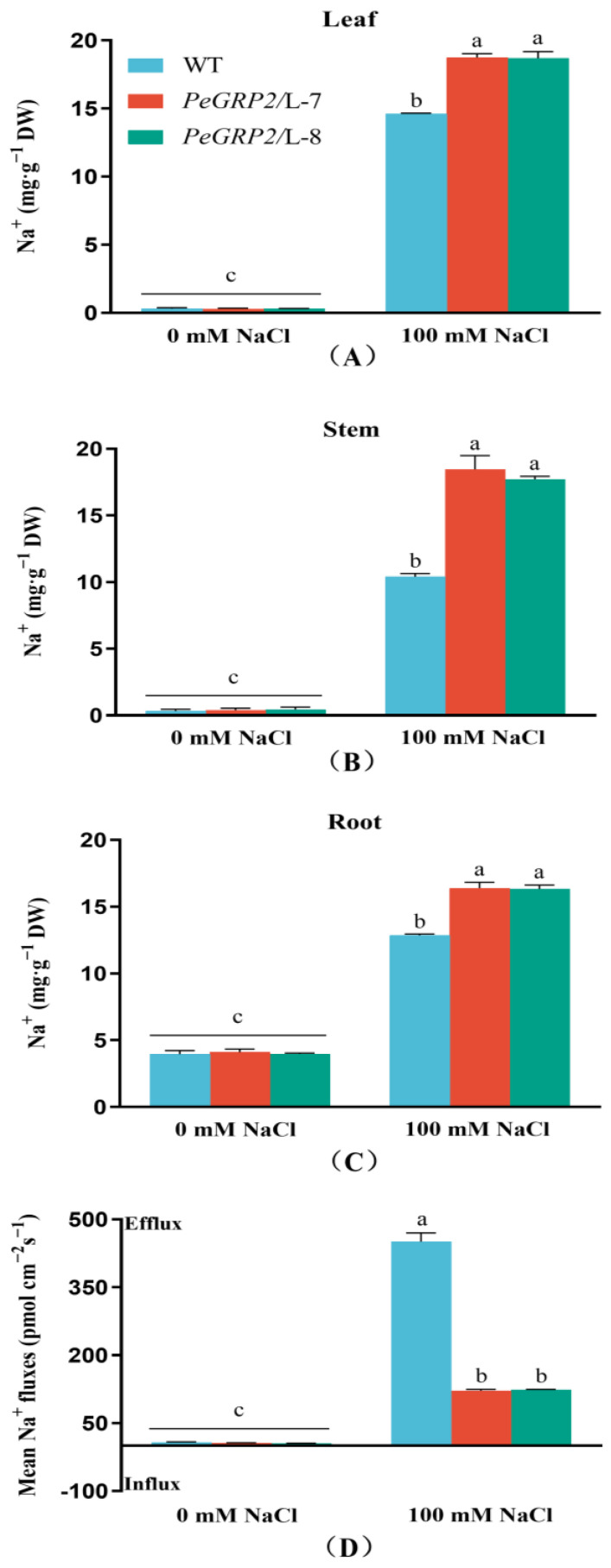
Na^+^ content and Na^+^ flux in wild-type *P.* × *canescens* and *PeGRP2*-overexpressing lines under long-term salt stress. (**A**) Na^+^ content in leaf. (**B**) Na^+^ content in stem. (**C**) Na^+^ content in root. (**D**) Na^+^ flux in root. The *PeGRP2*-overexpressing lines (L-7 and L-8) and wild-type (WT) *P. × canescens* were treated with NaCl saline (0 or 100 mM) for 15 days. The Na^+^ content in the roots, stems, and leaves and Na^+^ flux in the root tips were determined in the no-salt control and salinized poplars. Root Na^+^ flux was continuously recorded for 5–8 min in the meristematic region. Data are means ± SD (n = 3), and bars with different letters indicate significant differences (*p* < 0.05).

Na^+^ was found to accumulate in the roots, stems, and leaves of the NaCl-stressed poplars, with higher concentrations observed in the transgenic lines (Figure 6A–C). Flux recordings using Na^+^-selective microelectrodes showed that NaCl significantly increased the Na^+^ efflux despite a high flux rate in the WT poplars (Figure 6D). The reduced ability to excrete Na^+^ in transgenic lines would result in greater salt accumulation in the roots and subsequent transport to the shoots (Figure 6A–C).

### 2.8. PeGRP2-Interacting Target mRNAs in P. × Canescens

Our data showed that the *PeGRP2*-overexpressing lines differed from the WT in mediating photosynthesis, antioxidant protection, and Na^+^ homeostasis under salt stress. GRP proteins interact with target mRNAs and affect their stability under stress conditions [20]. To determine whether PeGRP2 regulates target mRNAs related to photosynthesis, antioxidant enzymes, and Na^+^ homeostasis, RNA affinity purification sequencing (RAP sequencing) was developed to enrich the target mRNAs that interacted with PeGRP2 in *P. × canescens*. Briefly, the PeGRP2-HaloTag protein was produced as a bait protein using the High-Yield Wheat Germ Protein Expression System, TnT SP6 [6]. The PeGRP2-HaloTag protein was incubated with Magne HaloTag magnetic beads to form a complex of the bait protein and magnetic beads. Then, the total RNA from the WT *P. × canescens* and *PeGRP2*-transgenic poplars was added to the bait protein–magnetic bead complex solution, forming a bait protein–mRNA–magnetic bead complex. The enriched mRNAs were eluted to construct a cDNA library for sequencing using the Illumina NovaSeq platform. The PeGRP2-interacting mRNAs encoding proteins related to photosynthesis, antioxidant defense, and Na^+^ homeostasis are listed in Table 1 and categorized into three groups, as follows.

1. Chloroplastic photosynthetic proteins: cytochrome b6-f complex iron-sulfur subunit (*PETC*), photosystem II 10 kDa polypeptide (*PSBR*), photosystem II core complex protein psbY (*PSBY*), photosystem II reaction center PSB28 protein (*PSB28*), chlorophyll a/b-binding protein 5 (*CAB5*), *CAB6*, ribulose-1, 5 bisphosphate carboxylase/oxygenase large subunit N-methyltransferase (*RBCMT*), light-harvesting complex-like protein 3 isotype 1 (*LIL3-1*), oxygen-evolving enhancer protein 1 (*PSBO1*), *PSBO2*, and ferredoxin-NADP reductase (*LFNR*).
2. Antioxidant enzymes: peroxidase 42 (*POD42*), *POD47*, superoxide dismutase (*SOD [Cu-Zn]2*), mitochondrial *SOD[Mn]*, transmembrane ascorbate ferrireductase 1 isoform X2 (*CYB1-2*), chloroplastic thioredoxin X (*TRX*), chloroplastic thioredoxin-like protein CDSP32 (*CDSP32*), chloroplastic thioredoxin M-type (*TRXM*), and chloroplastic peroxiredoxin Q (*PRXQ*).
3. Cation/H^+^ exchangers and ATPases: sodium/proton antiporter 1 (*NHA1*), sodium/hydrogen exchanger 2 isoform X1 (*NHE2-1*), vacuolar cation/proton exchanger 3 (*CAX3*), pyrophosphate-energized vacuolar membrane proton pump 1 (*AVP1*), plasma membrane (PM)-type ATPase 11, (*AHA11*), PM-type calcium-transporting ATPase 8 (*ACA8*), *ACA9*, AAA-ATPase At1g43910 (*AATP*), and mitochondrial AAA-ATPase ASD (*ASD*).

### 2.9. Transcriptional Profiling of the PeGRP2-Interacting mRNAs under Salt Stress

**Figure 7:**
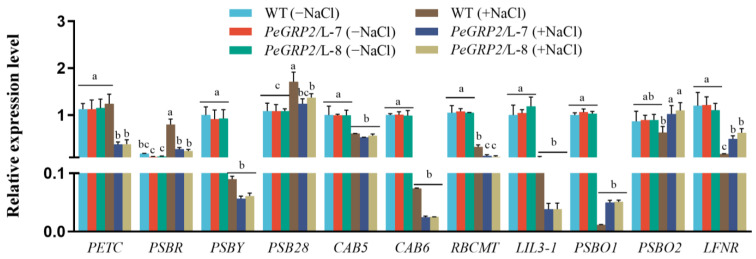
Transcript profiles of PeGRP2 target mRNAs encoding photosynthetic proteins in *P.* × *canescens* under long-term salt stress. The *PeGRP2*-overexpressing lines (L-7 and L-8) and wild-type (WT) *P. × canescens* were treated with NaCl saline (0 or 100 mM) for 15 days. Leaves were collected from no-salt control and salinized plants for RT-qPCR analysis. The PeGRP2 target mRNAs encoding chloroplastic photosynthetic proteins, such as cytochrome b6-f complex iron-sulfur subunit (*PETC*), photosystem II 10 kDa polypeptide (*PSBR*), photosystem II core complex protein psbY (*PSBY*), photosystem II reaction center PSB28 protein (*PSB28*), chlorophyll a/b-binding protein 5 (*CAB5*), *CAB6*, ribulose-1, 5 bisphosphate carboxylase/oxygenase large subunit N-methyltransferase (*RBCMT*), light-harvesting complex-like protein 3 isotype 1 (*LIL3-1*), oxygen-evolving enhancer protein 1 (*PSBO1*), *PSBO2*, and ferredoxin-NADP reductase (*LFNR*), were examined in WT and *PeGRP2*-overexpressing poplars. The primer sequences for PeGRP2-interacting mRNAs and the reference gene, *PcUBQ*, are shown in Supplementary Table S1. Data are means ± SD (n = 3), and bars with different letters indicate significant differences (*p* < 0.05).

**Figure 8:**
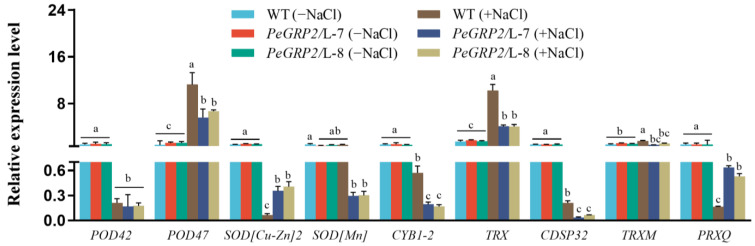
Transcript profiles of PeGRP2 target mRNAs encoding antioxidant enzymes in *P. × canescens* under long-term salt stress. The *PeGRP2*-overexpressing lines (L-7 and L-8) and wild-type (WT) *P. × canescens* were treated with NaCl saline (0 or 100 mM) for 15 days. Leaves were collected from no-salt control and salinized plants for RT-qPCR analysis. The PeGRP2 target mRNAs encoding antioxidant enzymes, such as peroxidase 42 (*POD42*), *POD47*, superoxide dismutase (*SOD[Cu-Zn]2*), mitochondrial *SOD[Mn]*, transmembrane ascorbate ferrireductase 1 isoform X2 (*CYB1-2*), chloroplastic thioredoxin X (*TRX*), chloroplastic thioredoxin-like protein CDSP32 (*CDSP32*), chloroplastic thioredoxin M-type (*TRXM*), and chloroplastic peroxiredoxin Q (*PRXQ*), were examined in WT and *PeGRP2*-overexpressing poplars. The primer sequences for PeGRP2-interacting mRNAs and the reference gene, *PcUBQ*, are shown in Supplementary Table S1. Data are means ± SD (n = 3), and bars with different letters indicate significant differences (*p <* 0.05).

**Figure 9:**
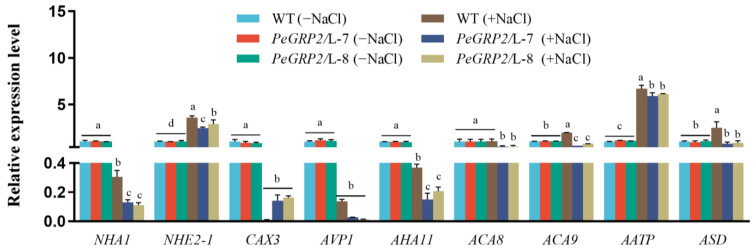
Transcript profiles of PeGRP2 target mRNAs encoding cation/H^+^ exchangers and ATPases in *P. × canescens* under long-term salt stress. The *PeGRP2*-overexpressing lines (L-7 and L-8) and wild-type (WT) *P. × canescens* were treated with NaCl saline (0 or 100 mM) for 15 days. Leaves were collected from no-salt control and salinized plants for RT-qPCR analysis. The PeGRP2 target mRNAs encoding cation/H^+^ exchangers and ATPases, such as sodium/proton antiporter 1 (*NHA1*), sodium/hydrogen exchanger 2 isoform X1 (*NHE2-1*), vacuolar cation/proton exchanger 3 (*CAX3*), pyrophosphate-energized vacuolar membrane proton pump 1 (*AVP1*), plasma membrane (PM)-type ATPase 11 (*AHA11*), PM-type calcium-transporting ATPase 8 (*ACA8*), *ACA9*, AAA-ATPase At1g43910 (*AATP*), and mitochondrial AAA-ATPase ASD (*ASD*), were examined in WT and *PeGRP2*-overexpressing poplars. The primer sequences for PeGRP2-interacting mRNAs and the reference gene, *PcUBQ*, are shown in Supplementary Table S1. Data are means ± SD (n = 3), and bars with different letters indicate significant differences (*p* < 0.05).

GRPs have been shown to modify the stability of target mRNAs under stress conditions [20]. Here, we used RT-qPCR to examine the transcripts of the PeGRP2-interacting mRNAs in the transgenic poplars after NaCl treatment (0 or 100 mM, 15 days). The PeGRP2 target mRNA transcripts were also tested in the WT and served as no-salt and salt controls. The NaCl-altered transcripts of the PeGRP2 target mRNAs in all tested lines are briefly listed below and shown in Figure 7, Figure 8 and Figure 9.

#### 2.9.1. Transcripts of the PeGRP2 Target mRNAs Encoding Photosynthetic Proteins

Under salt-free conditions, the transcripts of the PeGRP2-interacting mRNAs *PETC*, *PSBR*, *PSBY*, *PSB28*, *CAB5*, *CAB6*, *RBCMT*, *LIL3-1*, *PSBO1*, *PSBO2*, and *LFNR* in the transgenic lines (L-7 and L-8) were similar to those in the WT poplars (Figure 7). However, NaCl decreased the transcripts of these PeGRP2 target mRNAs, *PETC*, *PSBY*, *CAB5*, *CAB6*, *RBCMT*, *LIL3-1*, *PSBO1*, and *LFNR*, in the transgenic lines, with a few exceptions (*PSBR*, *PSB28*, and *PSBO2*; see Figure 7). Overall, most of the PeGRP2-interacting mRNAs involved in photosynthesis processes—for example, photosynthetic light-harvesting and reaction (*PSBY*, *CAB5*, *CAB6*, *LIL3-1*), oxygen-evolving (*PSBO1*), electron transport (*PETC* and *LFNR*), and carbon fixation (*RBCMT*)—were downregulated relative to the few unchanged target mRNAs under NaCl stress (Figure 7). This was consistent with the salt-reduced maximum photochemical efficiency of PSII, actual photosynthetic quantum yield, relative electron transport rate, and net photosynthetic rate in the transgenic poplars (Figure 4B,D–F). Compared to the plants with *PeGRP2* transgenes, the transcripts of the photosynthesis-related mRNAs were less reduced (*RBCMT*), unchanged (*PETC*), or even increased (*PSBR* and *PSB28*) in the WT after salt exposure (Figure 7). This was in line with the less restricted YII, Fv/Fm, ETR, and Pn values in the salt-stressed poplars of the WT (Figure 4B,D–F).

#### 2.9.2. Transcripts of the PeGRP2 Target mRNAs Encoding Antioxidant Enzymes

NaCl was shown to increase the transcripts of PeGRP2 target mRNAs such as *POD47* and *TRX* in the plants that overexpressed *PeGRP2* (Figure 8). However, the salt-elevated transcripts of *POD47* and *TRX* were typically lower in the *PeGRP2*-overexpressing poplars than in the WT (Figure 8). In contrast, NaCl decreased the transcripts of *POD42*, *SOD[Cu-Zn]2*, *SOD[Mn]*, *CYB1-2*, *CDSP32*, and *PRXQ* in the transgenic lines (Figure 8). In particular, the target mRNAs of PeGRP2, *SOD[Mn]*, *CYB1-2*, and *CDSP32* were more strongly suppressed by NaCl in the *PeGRP2* transgenic lines L-7 and L-8 compared to the WT (Figure 8). Meanwhile, NaCl’s suppression of the *SOD[Cu-Zn]2* and *PRXQ* transcripts was more pronounced in the WT (Figure 8). Our data suggest that PeGRP2 differentially regulates the stability of target mRNAs that encode antioxidant enzymes under salinity, differing from the no-salt controls, in which the transcripts of PeGRP2-interacting mRNAs remained at a similar level for all genotypes tested.

#### 2.9.3. Transcripts of the PeGRP2 Target mRNAs Encoding Cation/H^+^ Exchangers and ATPases

NaCl decreased the transcripts of PeGRP2-interacting mRNAs encoding the cation/H^+^ exchangers *NHA1* and *CAX3* but increased the *NHE2-1* transcript in the transgenic lines (Figure 9). Compared to the WT poplars, the *PeGRP2* transgenic plants exhibited lower levels of *NHA1* and *NHE2-1* under saline conditions (Figure 9). Therefore, PeGRP2 negatively regulates the stability of the target mRNAs encoding Na^+^/H^+^ antiporters under salt conditions.

The RT-qPCR analysis of the target mRNAs interacting with PeGRP2 showed that NaCl significantly decreased the transcripts of *AVP1*, *AHA11*, *ACA8*, and *ACA9* but increased the transcripts of *AATP* in the transgenic plants (Figure 9). Compared with the transgenic lines, NaCl produced a smaller decrease in the transcripts of *AHA11*, *ACA8*, and *ACA9* in the WT poplars, and *AATP* and *ASD* remained at a higher level under salt stress (Figure 9). Overall, the transcripts of all tested ATPase-encoding mRNAs were typically lower in the *PeGRP2*-overexpressing lines than in the WT, indicating that PeGRP2 negatively regulates the stability of the target mRNAs encoding ATPases under salt stress.

## 3. Discussion

### 3.1. PeGRP2 Increases the Salt Sensitivity of Transgenic P. × canescens

In this study, the overexpression of *P. euphratica PeGRP2* increased the sensitivity of *P.* × *canescens* to NaCl stress. Salt treatment resulted in the greater impairment of the shoot height, stem diameter, and leaf area in the *PeGRP2*-overexpressing poplars compared to the WT (Figure 3). Our data suggest that *PeGRP2* negatively regulates salt tolerance in poplars. This is consistent with the finding that the ectopic expression of *ZjGRP* or *MsGRP* inhibits seed germination and plant growth under salt treatment [48,49]. Similarly, AtGRP7 and AtGR-RBP4 negatively affect germination in salt media [33,50]. However, these results are inconsistent with the finding that AtGRP2 increases seed germination under salinity [19]. In addition, plant growth and flowering were promoted in lettuce plants overexpressing *AtGRDP2* [47]. These contrasting results indicate that GRP proteins play different roles in regulating the responses of plants to salinity. In agreement with this, CSDP1 and CSDP2 were also found to function in opposite ways in salt-exposed plants [54]. The GRP2 sequence analysis showed that PeGRP2 is homologous to *P. trichocarpa* PtGRP2 but distinct from *Arabidopsis* AtGRP2 (Figure 2), implying that the function of PeGRP2 is different from that of AtGRP2. The *PeGRP2* in the *P. euphratica* leaves tended to decrease after a transient increase in NaCl exposure after 3–6 h (Figure 1). Similarly, the *MhGR-RBP1* gene of *Malus hupehensis* increased after initial salinity but markedly decreased with a longer duration of stress [24]. The pattern of *PeGRP2* transcription contrasts with that of the salt-invoked *GRP* ortholog *SbGR-RNP* in *Sorghum bicolor* [46]. Thus, the downregulation of *PeGRP2* favors the adaptation of the salt-resistant poplar *P. euphratica* to saline conditions, as *PeGRP2* overexpression resulted in excessive Na^+^ accumulation and growth suppression in poplars (Figure 3 and Figure 6). PeGRP2’s suppression of salt tolerance is mainly due to the reduced ability to maintain photosynthesis, Na^+^, and ROS homeostasis in transgenic poplars (Figure 4, Figure 5 and Figure 6). We developed RNA affinity purification sequencing (RAP sequencing) to identify the target mRNAs that directly interacted with PeGRP2 in poplars (Table 1). Our RAP sequencing, in conjunction with RT-qPCR, revealed that PeGRP2 negatively regulated the stability of several target mRNAs encoding photosynthetic proteins, antioxidant enzymes, ATPases, and Na^+^/H^+^ transporters in the transgenic poplar under salt stress (Figure 7, Figure 8 and Figure 9).

### 3.2. PeGRP2 Interacts with Target mRNAs Encoding Photosynthetic Proteins and Affects Photosynthesis under Salt Stress

The reduction in the chlorophyll content and photosynthesis due to NaCl impaired the shoot and leaf growth of the poplars (Figure 3 and Figure 4). The salt-decreased photosynthetic capacity was accompanied by reduced transcripts of PeGRP2-targeting mRNAs encoding photosynthetic proteins involved in light-harvesting and reaction, electron transport, oxygen evolution, and CO_2_ fixation (Figure 7). NaCl significantly decreased the transcripts of *PETC*, *PSBY*, *CAB5*, *CAB6*, *RBCMT*, *LIL3-1*, *PSBO1*, and *LFNR*, which showed physical interactions with PeGRP2 in the transgenic poplars (Figure 7). The salt-reduced transcripts of the photosynthetic proteins resulted in reductions in the maximum photochemical efficiency of PSII, the actual photosynthetic quantum yield, the relative electron transport rate, and the net photosynthetic rate in the transgenic poplars (Figure 4). Compared with the plants transformed with *PeGRP2*, NaCl increased the transcripts of *PSBR* and *PSB28* in the WT poplars, and *PETC* and *RBCMT* showed less reduced or non-reduced transcripts after salt exposure (Figure 7). In accordance with this, YII, Fv/Fm, ETR, and Pn were less restricted in the WT poplars under salt conditions compared with the *PeGRP2*-overexpressing lines (Figure 4). We found that PeGRP2 decreased the transcripts of the target mRNAs irrespective of salt-simulated *PSB28* and *PSBR* and salt-inhibited *PETC* and *RBCMT* under salt stress (Figure 7). It is possible that NaCl changed the association of PeGRP2 with its target mRNAs [20], thereby negatively regulating the stability of the mRNAs and reducing photosynthesis in the transgenic poplars.

### 3.3. PeGRP2 Interacts with Target mRNAs Encoding Antioxidant Enzymes and Affects the ROS Scavenging Capacity under Salt Stress

NaCl caused the significant activation of POD, SOD, and CAT in the WT and transgenic poplars (Figure 5). Accordingly, NaCl increased the transcripts of several PeGRP2-interacting mRNAs, such as *POD47*, *TRX*, and/or *TRXM*, in the salt-stressed poplars, although the transcripts were typically lower in the *PeGRP2*-overespressing lines (Figure 8). However, salt repressed the transcripts of other PeGRP2-interacting mRNAs, i.e., *POD42*, *SOD[Cu-Zn]2*, *SOD[Mn]*, *CYB1-2*, *CDSP32*, and *PRXQ*, in the transgenic poplars (Figure 8). Compared with the *PeGRP2* transgenic lines, the *SOD[Mn]*, *CYB1-2*, and *CDSP32* transcripts were less reduced by salt in the WT (Figure 8). The lower stimulation of *POD47*, *TRX*, and *TRXM* together with the greater reduction in *SOD[Mn]*, *CYB1-2*, and *CDSP32* probably resulted in a less pronounced increase in the antioxidant enzyme activity in the transgenic poplar under salinity (Figure 5 and Figure 8). The impaired activity and transcripts of the antioxidant enzymes led to the failure to remove salt-induced ROS during long-term salt stress, which exacerbated the process of lipid peroxidation in the membranes. As a result, the REL and MDA content in the transgenic *P.* × *canescens* were remarkable higher than those in the WT poplar (Figure 3). In the leaves of *P. popularis*, we found that salt exposure increased the activity of APX, CAT, and GR [55,56]. However, the salt-induced production of ROS exceeded the antioxidant capacity of the enzymatic system, resulting in oxidative damage in the salt-sensitive poplars [55,56]. Our results are in agreement with the finding that *ZjGRP* overexpression resulted in downregulated *SOD* and *POD* in *Arabidopsis*, and transgenic plants exhibited salt-sensitive traits [49]. In contrast, *MpGR-RBP1* overexpression decreased the salt-produced ROS in *Arabidopsis* [52], and *LbGRP1*-overexpressing tobacco plants showed an increased proline content and activity of CAT and SOD under salt stress [51]. Apparently, GRPs regulate antioxidant defense in a species-specific manner. In our study, RAP sequencing and RT-qPCR showed that PeGRP2 interacted with the target mRNAs to negatively regulate their stability, independently of the salt-simulated *POD47* and *TRX* and salt-inhibited mitochondrial superoxide dismutase [Mn] (*SOD[Mn]*), *CYB1-2*, and *CDSP32* (Figure 8). The data from the RAP sequencing combined with the RT-qPCR are consistent with the results from RNA immunoprecipitation analysis and proteomic analysis. RNA immunoprecipitation analysis revealed that *OsGRP3* negatively regulated the expression of ROS regulatory genes in rice, such as metallothionein 1d (*MT1d*) and peroxidase 1 (*POX1*), under water-deficient conditions [20]. Proteomic analyses have shown that mitochondrial superoxide dismutase [Mn] and mitochondrial peroxiredoxin, whose function are related to controlling ROS homeostasis, are suppressed by AtGRP2 under cold stress [19]. Therefore, it is possible that PeGRP2 interacts with the target mRNAs of several antioxidant enzymes to negatively regulate their stability under salinity, resulting in a reduced ability to eliminate salt-generated ROS in transgenic poplars.

### 3.4. PeGRP2 Interacts with Target mRNAs Encoding ATPases and Na^+^/H^+^ Transporters and Affects Na^+^ Homeostasis under Salt Stress

In addition to affecting photosynthesis and antioxidant defense, PeGRP2 impairs ion homeostasis in salt-stressed poplar plants. The *PeGRP2*-overexpressing *P.* × *canescens* exhibited greater Na^+^ accumulation in its roots, stems, and leaves compared to the WT poplars (Figure 6). The buildup of Na^+^ in the shoots was due to the weak ability of the roots to excrete Na^+^ ions. The NMT data showed that the transgenic poplars had a significantly lower Na^+^ efflux at the root tips (Figure 6), indicating that a greater amount of salt ions absorbed by the roots was translocated to the shoots [57]. Our data are inconsistent with those for transgenic tobacco plants overexpressing *LbGRP*, in which the transgenic plants accumulated a lower Na^+^ content under saline conditions [51]. In general, excess Na^+^ leads to ion-specific toxicity and ROS production in poplar leaves [55,56]. Compared with the WT poplars, NaCl caused a greater reduction in the transcripts of *AHA11* and *NHA1*, which physically interact with PeGRP2, in the transgenic poplars (Figure 9). This could reduce the antiport of Na^+^/H^+^ across the PM in the leaf cells [57,58], although the transcripts of the PeGRP2-targeting mRNAs *AATP*, *NHE2-1*, and *ASD* were increased or unchanged by NaCl (Figure 9). Moreover, the downregulated transcripts of Ca^2+^-ATPase, *ACA8*, and *ACA9* in the transgenic poplars (Figure 9) suggest that PeGRP2 negatively regulates the stability of its target RNAs and thus impairs Ca^2+^ salt overly sensitive (SOS) signaling for Na^+^ extrusion under salt conditions. In accordance, the transgenic poplars showed downregulated transcripts of *SOS2* and *SOS3* under salt stress (Supplementary Figure S2). The vacuolar salt compartmentalization might also be impaired in transgenic lines, as a decrease in *AVP1* and *CAX3* transcripts was observed under salt stress (Figure 9). The reduced extrusion of Na^+^ from the cytosol and the accompanied restriction of vacuolar Na^+^ compartmentalization would result in an excess of toxic Na^+^ in the cytoplasm [58]. Consequently, the accumulated Na^+^ ions in the cytosol would lead to increased ROS production and decreased photosynthesis [55,56,59,60].

## 4. Materials and Methods

### 4.1. NaCl Treatment of P. euphratica

The *Populus euphratica* seedlings (1 year old) were obtained from the Xinjiang Uygur Autonomous Region of China. In April, the poplars were planted in 10 L individual containers filled with sandy soil (soil:sand = 1:1, *v*/*v*) and placed in a greenhouse at Beijing Forestry University. The *P. euphratica* seedlings were watered twice a week and fertilized with MS nutrient solution for three months in a greenhouse [55,56]. The light intensity (photosynthetically active radiation, PAR) in the greenhouse was 200–300 μmol m^−2^ s^−1^, with a 16 h photoperiod (7:00 AM–11:00 PM). The greenhouse temperature was maintained at 20–25 °C. Uniform seedlings were watered with NaCl solution (0 or 100 mM) for 12 days. The fine roots, shoots, and upper leaves (5th to 20th from the shoot tip) were collected on day 1 (0 h, 3 h, 6 h, 12 h, 24 h), day 4, day 7, and day 12. The samples were immediately frozen in liquid N_2_ and stored at −80 °C. Total RNA was isolated for the *PeGRP2* cloning and RT-qPCR analysis.

### 4.2. PeGRP2 Cloning and Sequence Analysis

The E.Z.N.A.^®^ Plant RNA kit (Omega Bio-Tek, Guangzhou, China) was used to isolate the total RNA from the *P. euphratica* leaves. After the removal of the genomic DNA using DNA remover mix, the reverse transcriptase kit HiFiScript gDNA Removal RT Master Mix (CoWin Biotech, Taizhou, China) was used for first-strand cDNA synthesis. *PeGRP2* was cloned via PCR amplification with the following specific primers: the forward primer 5′-ATGGCTGCCGAGGTTGAGTATA-3′ and the reverse primer 5′-CTAATCCCTCCAGCTACCAC-3′. The multiple sequence alignment of the GRP2 proteins was performed using CLUSTALW (http: //www.genome.jp/tools/clustalw/, accessed on 16 August 2022, EMBL-EBI, Hinxton, Cambridgeshire, UK). The phylogenetic tree was constructed using the MEGA11 software (http://www.megasoftware.net, accessed on 16 February 2023, the Center for Evolutionary Medicine and Informatics, Tempe, AZ, USA) [7]. The GenBank accession numbers of the GRP2 orthologs are listed in Supplementary Table S2.

### 4.3. PeGRP2 Transformation into P. × canescens

The transformation of *PeGRP2* into *P. × canescens* was performed according to a previous report but with modifications [61]. Briefly, the coding sequence of *PeGRP2* was ligated into the pART-CAM-FLAG vector, with the Xba I and Xho I sites driven by the cauliflower mosaic virus (CaMV) 35S promoter. Subsequently, the *PeGRP2* overexpression construct was transferred into *Agrobacterium tumefaciens* (strain GV3101) for plant transformation. A total of 10 lines overexpressing *PeGRP2*, i.e., L-1, L-2, L-3, L-4, L-5, L-6, L-7, L-8, L-9, and L-10, were obtained and verified using both semiquantitative RT-PCR and RT-qPCR.

Tissue cultures of stem segments in MS solid medium were used to propagate the plantlets [62]. They were cultivated in a climate incubator with the following settings: a temperature of 23 °C, relative humidity of 55–60%, a photoperiod of 16 h (light)/8 h (dark), and photoactive radiation of 150 μmol m^−2^ s^−1^. The *P. × canescens* plantlets were grown in tissue culture flasks for 3–4 weeks and then acclimated in hydroponics for 3 weeks before being planted into pots for soil culture. The nursery soil contained peat, silica sand, and potting soil at a ratio of 1:1:1. The plantlets were cultured in a climate-controlled room for four weeks and used for phenotype testing.

### 4.4. Phenotype Testing of Salt Tolerance

#### 4.4.1. Growth Measurement

Uniform plants of the wild-type (WT) *P.* × *canescens* and two transgenic lines, L-7 and L-8, were watered with NaCl saline (0 or 100 mM) for 15 days. The soil-cultured plants were watered twice a week. The shoot height, stem diameter, and leaf area growth of the developing leaves were measured on day 1, day 10, and day 15. For the WT and two transgenic lines, six individual plants were set up for each treatment, the control and salt treatment.

#### 4.4.2. Relative Electrolyte Leakage (REL) and Malondialdehyde (MDA) Content

The upper leaves (3rd to 8th from the top) were collected from the WT *P. × canescens* and two *PeGRP2*-overexpressing lines, L-7 and L-8, after 15 days of NaCl treatment (0 or 100 mM). The REL was calculated from the initial relative conductivity (EC1) before boiling and the final conductivity (EC2) after boiling: REL (%) = (EC1/EC2) × 100% [7]. The MDA content was determined using the micro MDA assay kit (BC0025) (Beijing Solarbio Science & Technology, Beijing, China). Fresh leaves (0.1 g) were ground into a homogenate by adding 1 mL of extraction buffer and then centrifuged at 8000× *g* for 10 min at 4 °C, and the supernatant was collected. The absorbance values (ΔA) were measured at 532 nm and 600 nm using a microplate reader. The MDA concentration (nmol g^−1^) was calculated as 32.258 × (ΔA_532_ − ΔA_600_)/0.1.

#### 4.4.3. Measurement of the Chlorophyll Fluorescence and Gas Exchange

The chlorophyll content, chlorophyll fluorescence, and gas exchange were measured after the WT poplar and *PeGRP2* transgenic lines were treated with NaCl (0 or 100 mM) for 10 and 15 days. The chlorophyll content of the upper mature leaves (3rd to 8th from the top) was measured using a portable chlorophyll meter, the SPAD-502Plus (Konica Minolta Optics, Tokyo, Japan). The maximum PSⅡ photochemical efficiency (Fv/Fm), the actual photosynthetic quantum yield (YⅡ), and the relative electron transport rate (ETR) were examined using a pulse-amplitude-modulated (PAM) chlorophyll fluorometer, the JUNIOR-PAM (Heinz Walz GmbH, Effeltrich, Germany). The net photosynthetic rate (Pn) and stomatal conductance (Gs) of the upper mature leaves (6th to 8th from the top) were measured using a portable open gas exchange system, the LI-6400 (Li-Cor, Inc., Lincoln, NE, USA).

### 4.5. Determination of the Antioxidative Enzyme Activity

The WT *P. × canescens* and two *PeGRP2*-overexpressing lines, L-7 and L-8, were salinized for 15 days with NaCl (0 or 100 mM). The leaves (3rd to 8th from the top) were sampled and used to measure the total activity of antioxidative enzymes. The POD, SOD, and CAT activity was examined using assay kits for POD (BC0090), CAT (BC0205), and SOD (BC0175) (Beijing Solarbio Science & Technology, Beijing, China), respectively.

### 4.6. Na^+^ Concentration in the Roots, Leaves, and Stems

Roots, stems, and leaves were collected from the soil-cultured WT *P.* × *canescens* and *PeGRP2*-overexpressing lines, L-7 and L-8, after 15 days of salt treatment (0 or 100 mM NaCl). The oven-dried samples (60 °C, 5 days) were digested with H_2_SO_4_-H_2_O_2_ and used for Na^+^ determination using an atomic absorption spectrometer (Varian SpectrAA 220FS, Palo Alto, CA, USA).

### 4.7. Flux Records of Na^+^ in the Roots

The net Na^+^ flux at the root tips was recorded using a non-invasive micro-test system (NMT) [57]. The roots were collected from the WT *P. × canescens* and *PeGRP2*-overexpressing lines, L-7 and L-8, after 15 days of salt treatment (0 or 100 mM NaCl). The root tips were equilibrated for 30 min in measuring solution (0.1 mM NaCl, 0.1 mM CaCl_2_, 0.1 mM MgCl_2_, and 0.5 mM KCl, pH 5.7). The selective microelectrodes for Na^+^ were calculated and used to monitor the net flux of Na^+^ in the apical meristem (300 μm from the root tip). Continuous recordings were made at each measuring point for 5–8 min, and the average flux at each point was calculated. Three to four individual plants of each genotype were used for the flux recording.

### 4.8. RNA Affinity Purification Sequencing

The RNA affinity purification sequencing (RAP sequencing) was performed with reference to the DNA affinity purification sequencing (DAP sequencing) protocol as described previously [6], but with modifications. In brief, the full-length *PeGRP2* was ligated into the pFN19K (HaloTag) T7 SP6 Flexi vector (Promega, Madison, WI, USA). The HaloTag-PeGRP2 protein was produced using the TnT SP6 High-Yield Wheat Germ Protein Expression System (Promega, USA) as a bait protein. The expression of the PeGRP2 protein was determined using Western blotting and purified using Magne HaloTag Beads (Promega, USA). Total RNA was extracted from the leaves of the WT *P. × canescens* and *PeGRP2* transgenic lines using the EASYspin Plus Kit (Aidlab, Beijing, China). Subsequently, the mRNA was enriched using the Hieff NGS mRNA Isolation Master Kit (Yeasen, Shanghai, China). The covalently conjunct beads and HaloTag-PeGRP2 protein were incubated with mRNA from the WT or transgenic poplars in binding buffer (10 mM HEPES, 1 mM DTT, 1 mM MgCl_2_, 20 mM KCl, pH 7.3). The bait protein was not added to the negative control but subjected to the same procedures as introduced for the HaloTag-PeGRP2 protein. The beads were washed with washing buffer (10 mM HEPES, 1 mM DTT, 1 mM MgCl_2_, 0.1% Tween 20, 20 mM KCl, pH 7.3). Finally, the mRNA was eluted from the beads with nuclease-free water. The cDNA library was constructed using the RNA-Seq Library Prep kit (Illumina, San Diego, CA, USA), followed by sequencing using the Illumina NovaSeq platform. The *P. × canescens* sequenced genome (sPta717alba.fasta.gz, https://www.aspendb.org/downloads, accessed on 28 July 2023) was used as the reference genome. To identify the PeGRP2-binding mRNAs, we used fastp software version 0.21.0 to filter reads [63]. All clean reads were mapped using hisat2 version 2.1.0 to the reference genome [64]. Moreover, the enriched mRNAs were analyzed using the software featureCounts, version 2.0.4 [65].

### 4.9. Semiquantitative RT-PCR and RT-qPCR Analysis

Semiquantitative RT-PCR was performed with the following specific primers for *PeGRP2*: the forward primer 5′-ATGGCTGCCGAGGTTGAGTATA-3′ and the reverse primer 5′-CTAATCCCTCCAGCTAC CAC-3′. The synthesized cDNA of the transgenic lines and non-transgenic lines was used as a template, and *PeActin7* served as an internal control [7]. The PCR running conditions for semiquantitative RT-PCR were as follows: 94 °C for 5 min, followed by 35 cycles of denaturation at 94 °C for 30 s, 55 °C for 30 s, extension at 72 °C for 1 min, and finally at 72 °C for 10 min. Then, the reaction product was resolved using electrophoresis on 1.2% (*w*/*v*) agarose gel stained with ethidium bromide. The gel images were captured using a Gel Doc 1000 DNA gel analysis and documentation system (Bio-Rad Laboratories, Hercules, CA, USA).

RT-qPCR was used to determine the transcription of *PeGRP2* in the roots, shoots, and leaves of *P. euphratica* during the period of salt treatment (0 or 100 mM, 12 days). The transcripts of the PeGRP2-targeting mRNAs in the transgenic *P. × canescens* were examined under the control and NaCl treatments (100 mM, 15 days). The transcripts of the PeGRP2-interacting mRNAs were also tested for the WT *P.* × *canescens* and served as non-salt and salt controls. RNA isolation from *P. euphratica* and *P.* × *canescens* was performed using the R6827 Plant RNA Kit (Omega Bio-Tek, Norcross, GA, USA). Then, the cDNA was synthesized via reverse transcription using HiFiScript gDNA Removal RT Master Mix (CoWin Biotech, Taizhou, China) as a template for RT-qPCR. The reaction system was prepared according to the UltraSYBR Mixture (Low ROX) (Beijing ComWin Biotech, Beijing, China) and monitored in real time using the LineGene 9600 Plus (Bioer Technology, Hangzhou, China). *PeActin7* and *PcUBQ* served as internal controls for *P. euphratica* [7] and *P.* × *canescens* [66]. The PCR running conditions for RT-qPCR were as follows: 95 °C for 10 min, followed by 35 cycles of denaturation at 95 °C for 10 s, 55 °C for 30 s, extension at 72 °C for 30 s, and finally at 72 °C for 10 min. Three individual biological replicates were set up for each treatment.

### 4.10. Data Analysis

The Na^+^ flux was calculated using the NMT flux rate conversion table in JCal v3.3 from Xuyue (http://www.xuyue.net/, accessed on 11 May 2022). All experimental data were statistically analyzed using SPSS version 19.0 (IBM Corporation, Armonk, NY, USA). The one-way ANOVA method was used to compare means between treatments. For post hoc multiple comparisons, the least significant difference (LSD) method was used. *p <* 0.05 or *p <* 0.01 was considered to indicate a significant difference, unless otherwise stated.

## 5. Conclusions

In conclusion, *P. euphratica* PeGRP2 negatively regulates the salt tolerance in poplars. The overexpression of *PeGRP2* in *P. × canescens* resulted in its reduced ability to maintain photosynthesis, antioxidant protection, and Na^+^ homeostasis under salt stress. NaCl decreased the transcripts of PeGRP2-interacting mRNAs encoding photosynthetic proteins, antioxidative enzymes, ATPases, and Na^+^/H^+^ antiporters in the transgenic poplars. We observed that PeGRP2 exerted negative effects on the stability of the target mRNAs under saline conditions, particularly those encoding the photosynthetic proteins *PETC* and *RBCMT*; antioxidant enzymes *SOD[Mn]*, *CDSP32*, and *CYB1-2*; ATPases *AHA11*, *ACA8*, and *ACA9*; and the Na^+^/H^+^ antiporter *NHA1*, resulting in a decreased ability to tolerate salinity stress in the transgenic *P.* × *canescens*. Accordingly, the downregulation of *PeGRP2* contributes to salt adaptation in the salt-resistant poplar *P. euphratica* during prolonged salt exposure. Therefore, this study provides new insights for the breeding of salt-resistant poplars by reducing the *GRP2* transcripts.

## Figures and Tables

**Table 1:** PeGRP2-interacting mRNAs in transgenic *P. × canescens*.

Accession Number	Description	Abbr.	Gene ID	Log2 (Fold_Change)
	Photosynthetic proteins			
XP_002319934.1	cytochrome b6-f complex iron-sulfur subunit, chloroplastic	*PETC*	Potri.013G148900	4.65
XP_002317015.1	photosystem II 10 kDa polypeptide, chloroplastic	*PSBR*	Potri.011G142200	4.96
XP_002315645.1	photosystem II core complex protein psbY, chloroplastic	*PSBY*	Potri.010G052000	1.92
XP_002303109.2	photosystem II reaction center PSB28 protein, chloroplastic	*PSB28*	Potri.002G256400	2.32
XP_002301582.1	chlorophyll a/b-binding protein 5, chloroplastic	*CAB5*	Potri.002G221400	3.45
XP_002315298.1	chlorophyll a/b-binding protein 6, chloroplastic	*CAB6*	Potri.010G221100	3.48
XP_024441106.1	ribulose-1, 5 bisphosphate carboxylase/oxygenase large subunit N-methyltransferase, chloroplastic	*RBCMT*	Potri.014G169300	1.57
XP_006368947.2	light-harvesting complex-like protein 3 isotype 1, chloroplastic	*LIL3-1*	Potri.001G151300	1.24
XP_002310188.1	oxygen-evolving enhancer protein 1, chloroplastic	*PSBO1*	Potri.007G033700	2.91
XP_002300858.1	oxygen-evolving enhancer protein 2, chloroplastic	*PSBO2*	Potri.002G055700	2.69
XP_006383096.1	ferredoxin-NADP reductase, leaf isozyme, chloroplastic	*LFNR*	Potri.005G112900	2.70
	Antioxidant enzymes			
XP_002304909.1	peroxidase 42	*POD42*	Potri.004G015300	2.25
XP_024448088.1	peroxidase 47	*POD47*	Potri.018G136900	2.77
XP_002325843.1	superoxide dismutase [Cu-Zn] 2	*SOD[Cu-Zn]2*	Potri.019G035800	2.04
XP_002319332.2	superoxide dismutase [Mn], mitochondrial	*SOD[Mn]*	Potri.013G092600	1.00
XP_024438027.1	transmembrane ascorbate ferrireductase 1 isoform X2	*CYB1-2*	Potri.012G141000	5.76
XP_002310066.2	thioredoxin X, chloroplastic	*TRX*	Potri.007G074000	1.02
XP_002307752.2	thioredoxin-like protein CDSP32, chloroplastic	*CDSP32*	Potri.005G245700	2.85
XP_002306676.1	thioredoxin M-type, chloroplastic	*TRXM*	Potri.005G186800	4.54
XP_002308370.2	peroxiredoxin Q, chloroplastic	*PRXQ*	Potri.006G137500	2.66
	Cation/H^+^ exchangers and ATPases			
XP_002298746.1	sodium/proton antiporter 1	*NHA1*	Potri.001G301000	2.49
XP_002307194.2	sodium/hydrogen exchanger 2 isoform X1	*NHE2-1*	Potri.005G045100	2.95
XP_002323578.2	vacuolar cation/proton exchanger 3	*CAX3*	Potri.016G115500	2.73
XP_006382405.2	pyrophosphate-energized vacuolar membrane proton pump 1	*AVP1*	Potri.005G018700	1.43
XP_024438330.1	ATPase 11, plasma membrane-type	*AHA11*	Potri.012G071600	1.01
XP_024459503.1	calcium-transporting ATPase 8, plasma membrane-type	*ACA8*	Potri.018G139800	2.02
XP_024466795.1	calcium-transporting ATPase 9, plasma membrane-type	*ACA9*	Potri.010G250800	1.66
XP_024461157.1	AAA-ATPase At1g43910	*AATP*	Potri.007G019600	1.10
XP_024455649.1	AAA-ATPase ASD, mitochondrial	*ASD*	Potri.004G012500	1.94

The PeGRP2-interacting mRNAs in *P.* × *canescens* were identified using RNA affinity purification sequencing. The database used for mRNA identification was AspenDB (https://www.aspendb.org, accessed on 20 July 2023). Accession number: the corresponding protein number in NCBI (https://www.ncbi.nlm.nih.gov, accessed on 20 July 2023). Gene ID: the ID number corresponding to the mRNA in the database. Log2(Fold_change): an estimate of the log2 ratio of mRNA enrichment in *PeGRP2*-overexpressing lines (L-7, L-8) to that in wild-type poplar.

## Data Availability

The data presented in this study are available in the article and Supplementary Materials.

## Supplementary Materials

The following supporting information can be downloaded at https://www.mdpi.com/article/10.3390/ijms25042046/s1.

## Abbreviations

AATP: AAA-ATPase; ACA8, 9: plasma membrane-type calcium-transporting ATPase 8, 9; AHA11: plasma membrane-type ATPase 11; ASD: mitochondrial AAA-ATPase; AVP1: pyrophosphate-energized vacuolar membrane proton pump 1; CAB5, 6: chlorophyll a/b-binding protein 5, 6; CAT: catalase; CAX3: vacuolar cation/proton exchanger 3; CDSP32: chloroplastic thioredoxin-like protein CDSP32; CYB1-2: transmembrane ascorbate ferrireductase 1 isoform X2; ETR: relative electron transport rate; Fv/Fm: maximum PSⅡ photochemical efficiency; Gs: stomatal conductance; GRP: glycine-rich RNA-binding protein; LFNR: chloroplastic ferredoxin-NADP reductase; LIL3-1: chloroplastic light-harvesting complex-like protein 3 isotype 1; NHE2-1: sodium/hydrogen exchanger 2 isoform X1; NMT: non-invasive micro-test; PETC: chloroplastic cytochrome b6-f complex iron-sulfur subunit; PM: plasma membrane; Pn: net photosynthetic rate; POD42, 47: peroxidase 42, 47; PRXQ: chloroplastic peroxiredoxin Q; PSB28: photosystem II reaction center PSB28 protein; PSBO1, 2: oxygen-evolving enhancer protein 1, 2; PSBR: photosystem II 10 kDa polypeptide; PSBY: photosystem II core complex protein psbY; RAP sequencing: RNA affinity purification sequencing; RBCMT: ribulose-1, 5 bisphosphate carboxylase/oxygenase large subunit N-methyltransferase; REL: relative electrolyte leakage; ROS: reactive oxygen species; RT-qPCR: real-time quantitative PCR; SOD[Cu-Zn]2: superoxide dismutase [Cu-Zn] 2; SOD[Mn]: mitochondrial superoxide dismutase [Mn]; SOS: salt overly sensitive; TRX: chloroplastic thioredoxin X; TRXM: chloroplastic thioredoxin M-type; YⅡ: actual photosynthetic quantum yield.
