# Supplementary material for: Populus euphratica GRP2 Interacts with Target mRNAs to Negatively Regulate Salt Tolerance by Interfering with Photosynthesis, Na+, and ROS Homeostasis
Source: Int J Mol Sci. 2024 Feb 7;25(4):2046. doi: 10.3390/ijms25042046 (PMC10888501; doi:10.3390/ijms25042046)
Supplement: Supplementary file 1 [file ijms-25-02046-s001.zip › ijms-2804587-supplementary.pdf]

Supplementary Table S1. Primers used for qRT-PCR.

Gene name	*Primer sequences (5'-3')*	
PeGRP2	*F: ATTACAGTGACGAGGACCACC*	*R: GGAAGGGACAAACGGAGACT*
PeActin7	*F: ATTGGCCTTGGGGTTAAGAG*	*R: CACACTGGAGTGATGGTTGG*
PcUBQ	*F: AGACCTACACCAAGCCCAAGAAGAT*	*R: CCAGCACCGCACTCAGCATTAG*
PcPETC	*F: ATTTCTTTGTCCCTTCTGGAC*	*R: GTTCCATGGCACAACACAC*
PcPSBR	*F: AACCTGAGGAACGGGTTA*	*R: AAGGATTCCAGCTAGGGTAA*
PcPSBY	*F: TACCACTCATACCAGCCATA*	*R: ATAGAATAGCAGGAGCAACG*
PcPSB28	*F: CCTACTCCCTATTCAATGGC*	*R: AAGGTGAATATCGCCATACC*
PcCAB5	*F: CACTTGGATGTGTCTTTCCT*	*R: CTGTACCCTTCAACGAATCC*
PcCAB6	*F: CCATTGAATTCGTTGCCATT*	*R: ACCAGGATAAGCTGTTTGTT*
PcRBCMT	*F: CTCTTTTGTACTGGACACGA*	*R: GACCACTTGAAAGTCTCCAT*
PcLIL3-1	*F: CTGCAATGATTGGGTTCTTC*	*R: TAGGGATGGAAGATGTCTCA*
PcPSBO1	*F: TCACCATTAAACAGTTGGTTG*	*R: AGGGTGATTTTACCTGTTGA*
PcPSBO2	*F: AACCTTTCTGTCATGGTCAC*	*R: TATTGTTTCCCATCGATCAC*
PcLFNR	*F: CTTGGAACTGGAACTGGTAT*	*R: TTTGTTTGCTCTCTACTGACT*
PcPOD42	*F: CTATCTCCCAGACCACAATG*	*R: CCTCACATATTGGACAGCTT*
PcPOD47	*F: CAAAAATAGGTTGAGTGACCC*	*R: GTTCATAGCGTAGCCCTT*
PcSOD[Cu-Zn]2	*F: TCATCACCGGAGACTCTAAT*	*R: CGTTCATTATCACATGGAGC*
PcSOD[Mn]	*F: AATCTCACTCCTGTCCAAGAA*	*R: TGGTCTCAACCACAAGTTTC*
PcCYB1-2	*F: GGAGGTGAAGCCATTATGAG*	*R: CCAGGGTAGAAGAACACAAT*
PcTRX	*F: TATGGGGATAGATTGACGGT*	*R: CTCCAAGAGAGCATCAACAT*
PcCDSP32	*F: AAGCTGATGGGAGATGTTTT*	*R: CTATCATTCTCGTCACCGTT*
PcTRXM	*F: AGTGAGTACTACCACCAGAG*	*R: ATCTGTCGCTACTTGAACTG*
PcPRXQ	*F: CTTTGCTTCCCACTCTTAAA*	*R: ACAAAGTCTTTCCATCCTGA*
PcNHA1	*F: GCTATACACACTTCAGGGAG*	*R: AAGCAGATAAGCACCAAGAA*
PcNHE2-1	*F: GAGATTGCAAATGTTGACCA*	*R: AGATAACAACCCCAGTACAC*
PcCAX3	*F: GTTTGTGGTTCCACTTTGTG*	*R: GTTTAAGAAAAAGCACGCCC*
PcAVP1	*F: GGATCAATACTGTCGATGTCT*	*R: TGGCTCTACCTTCCATAAGT*
PcAHA11	*F: TATTTGGAGTGTCAACCCTG*	*R: GAGCAATGATAAAAGCCACC*
PcACA8	*F: TGAGTTTGTTGGGAAGTTCA*	*R: CGATCACTTGGAGAGTTACC*
PcACA9	*F: ATGAGTAGCAGTTGTTCGTC*	*R: CGAAGAATCTCGATAGGAGC*
PcAATP	*F: TGTCCTTTTCAAATGCGTTG*	*R: TGGATTGTTTCACATCCTGT*
PcASD	*F: ATAAAGGACAACAGCGAGTT*	*R: CTTTCTTCTTCTGCCTCCTT*

Supplementary Table S2. Accession numbers of GRP orthologs used in multiple sequence alignment and phylogenetic analysis.

Sequence name	Accession number
PeGRP2	XP_011046516.1
PtGRP2	XP_024463774.1
GmGRP2	XP_003540832.1
MdGRP2	XP_008338784.2
OsGRP2	XP_015649739.1
ZmGRP2	ACN27139.1
NtGRP2	XP_016516220.1
CsGRP2	XP_010428999.1
AtGRP2	NP_179702.1
SbGR-RNP	AAG23220.1
OsGRP3	BAS81800.1
AtGRP7	NP_179760.1
MhGR-RBP1	HM042682.1
ZjGRP	AIN39847.1
AtGR-RBP4	NP_189025.1
LbGRP1	ACV33079.1

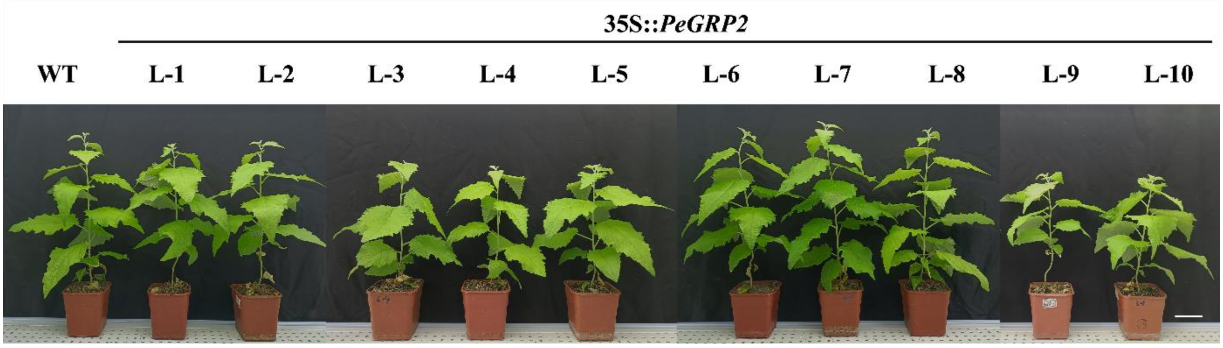

Supplementary Figure S1. Plant growth of wild-type *P.* × *canescens* and *PeGRP2*-overexpressing lines (L-1, L-2, L-3, L-4, L-5, L-6, L-7, L-8, L-9, and L-10). Tissue cultures of stem segments in MS solid medium were used to propagate the plantlets. *P.* × *canescens* plantlets grown in tissue culture flasks for 3-4 weeks were acclimated in hydroponics for 3 weeks before planting in individual pots for soil culture. The nursery soil contained peat, silica sand, and potting soil in a ratio of 1:1:1. Then the plantlets were transferred to a climate-controlled room for 4 weeks culture. The light intensity (photosynthetically active radiation, PAR) was 200-300 μmol m^{-2} s^{-1} with a 16-h photoperiod (7:00 AM–11:00 PM). The room temperature was maintained at 20-25 °C. The plants were watered twice a week and representative images are shown. Scale bars = 5 cm.

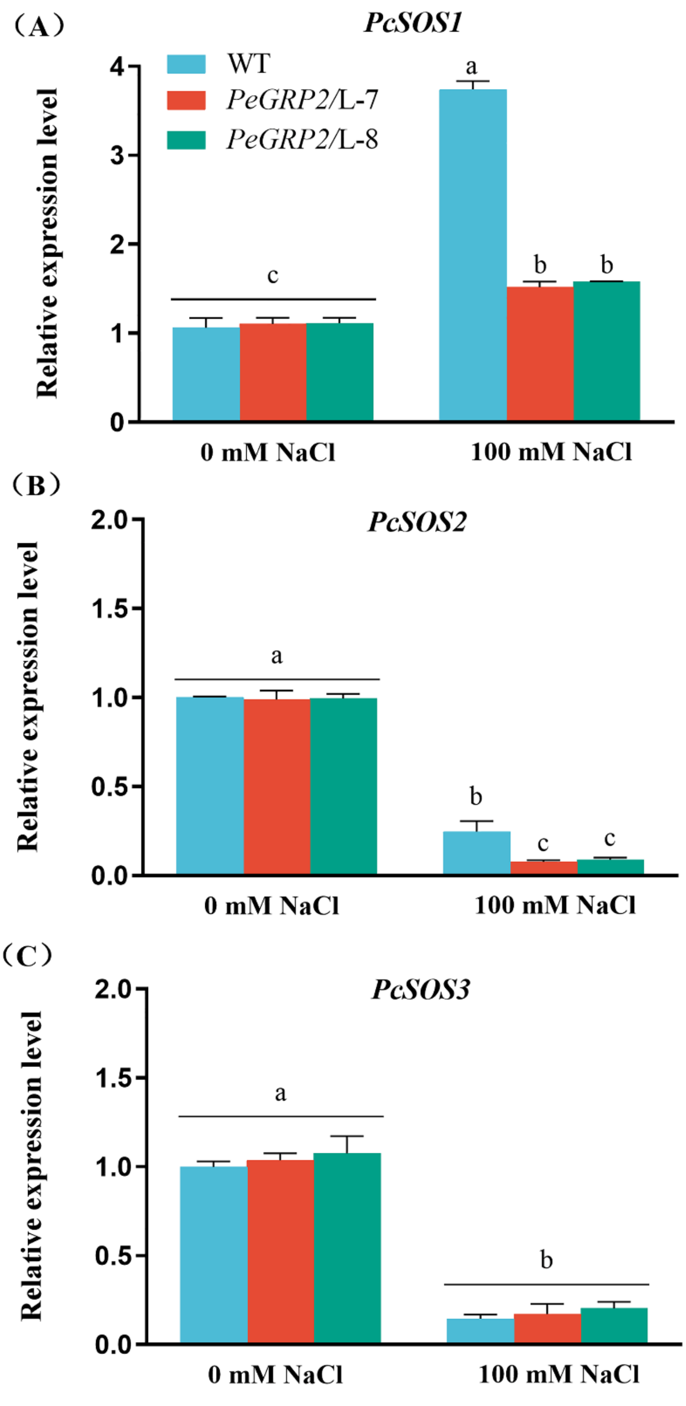

Supplementary Figure Figure S2. Transcript of *PcSOS1*, *PcSOS2* and *PcSOS3* in *PeGRP2*-overexpressing *P.* × *canescens* under long-term salt exposure. The wild-type (WT) *P.* × *canescens* and *PeGRP2*-overexpressing lines (L-7 and L-8) were treated with NaCl saline (0 or 100 mM) for 15 days. Leaves were collected from no-salt control and salinized plants for RT-qPCR analysis. Transcripts of salt-overly-sensitive (SOS) pathway genes *PcSOS1*, *PcSOS2* and *PcSOS3*, were examined in WT and *PeGRP2*-overexpressed poplars. The primer sequences for SOS and reference genes, *PcUBQ*, are shown in Supplementary Table S1. Data are mean ± SE (n = 3), and different letters indicate significant difference (P < 0.05).
